# Supplementary material for: Why women with breast cancer presented late to health care facility in North-west Ethiopia? A qualitative study
Source: PLoS One. 2020 Dec 4;15(12):e0243551. doi: 10.1371/journal.pone.0243551 (PMC7717512; doi:10.1371/journal.pone.0243551)
Supplement: S1 File — (PDF) [file pone.0243551.s001.pdf]

I. Study Information Sheet (SIS) for in participants of in-depth interview

Greeting; good morning/afternoon!

Hello! dear participant!

We are carrying out a study on "why women with breast cancer presented late to health care facility in North-west Ethiopia?" Therefore inorder to achive this objective having an in-depth interview with you has become very important. We hope that the interview we would be having with you is very much helpful for the community and government bodies to explore the reasons for late presentation of patients with breast cancer care to make intervetions related with early detection, diagnosis and management of breast cancer by developing different intervention startegies and polices. We would like to tell you that you are selected purposely to be participant of this Study.

Objective of the study: To explore the reasons for late presentation to medical care for breast cancer in North West Ethiopia.

Benefit of the study: The result will provide important information on the different reasons of breast cancer patients for delay in presentation to the health care system and to take appropriate intervention on the problem based on the findings of the study.

Risk of the study: Participating in this study will not have any risk or harm.

Costs to the participants: Your participation in this research will not cost you anything.

Rights of Participation: Your participation in this research is entirely of your own free will and you are free to withdraw at any time during the course of the study without offering any reasons why. You may respond to all the questions or you may not answer to questions you do not want to and you may end the interview at any time you want. Withdrawal from this study will not affect your care in this hospital in anyway. You can ask any question that is not clear for you.

Confidentiality: All information provided in this study will be confidential. All forms will be coded and information will be entered into password protected computers. We will do all that is in power to ensure that your identity and the information that you have provided is kept confidential.

II. Informed consent form for study participants

As to the information given above, Participation in this in-depth interview is voluntary and has no any risk. Your answers will remain confidential, and we will not be taking down your name or address, so your answers will be anonymous. You can choose not to answer any individual question that you do not want to answer and you may end this interview at any time you want. However, we hope that you will participate in this in depth interview since your honest response to the interviews will be very important for the purpose of the study.

At the same time we would like to appreciate your voluntarily participation in the study after a thorough understanding of the information given to you.

Now, are you willing to participate in this study?

1. No (say thank you)
2. Yes (continue interviewing)

Name of principal investigator: Aragaw Tesfaw

Cell phone: 0921743820

Email: aragetesfa05@gmail.com

Name of health facility__________________________

Name of interviewer_______________________ signature__________

Date of interview (E.C) _____/_____/_______

In doing this interview, we will raise some questions concerning why women with breast cancer come late to health facility?

III. **In-depth interview Guide (discussion points with breast cancer patients) at University of Gondar and Felege Hiwot comprehensive specialized hospitals in North west Ethiopia.**

1. Would you please tell me your age, educational status, occupation, marital status and how long you have been diagnosed with breast cancer? No need to mention your name.
2. How much time you delay from your first symptom recognition until your first medical visit? why not you came to health facility immediately when you see the initial breast abnormalites? Why you did not you seek early medical care/ what things make you late?

   Probing questions
   - Personal related barriers?
   - Cultural factors?
   - Family related factors?
   - Religious belifs?
   - Information access about breast cancer related barriers?.
3. In your opinion, what do you understand about breast cancer before?

   Probing questions
   - Perception about severity of the disease?
   - preventability, risk factors,
   - clinical features
   - knowledge and practice of Breast self examination, clinical breast examination by health care provider?
4. What did you feel when you notice changes in your breast?

   Probing questions
   - Do you think that it will be cancer?
   - To whom you first told your problem?
   - What solutions you made to manage the abnormality in your breast?
   - , did you use traditional treatments, holy water?
5. In your opinion why other women come late to health facility after the disease is advanced?

Probing questions

- Personal related barriers?
- Cultural factors?
- Family related factors?
- Religious belifs?
- Information access about breast cancer related barriers?.
- How the community sees breast cancer?
- Perception to the disease, severity, stigma and discrimination, support to patients.
- how all these will affect patients not to seek early medical care?

6. Do you have history of practicing breast cancer early detction methods?

Probing questions

- Breast self-examination?
- Clinical breast examination?
- Mamography?

7. Do you have anything to share me which is not raised on the discussion before I end the interview?

Finally, I would like to express my heartfelt thanks for your voluntary participation in this in-depth interview.
