# Supplementary material for: Why women with breast cancer presented late to health care facility in North-west Ethiopia? A qualitative study
Source: PLoS One. 2020 Dec 4;15(12):e0243551. doi: 10.1371/journal.pone.0243551 (PMC7717512; doi:10.1371/journal.pone.0243551)
Supplement: S2 File — (PDF) [file pone.0243551.s002.pdf]

Table 2: Themes and subthemes explored from breast cancer patients at University of Gondar and Felege Hiwot Specialized hospitals, North-west Ethiopia, 2019.

Themes	Narrations from participants (main findings)
**Lack of knowledge and awareness about breast cancer**	*"I do not know about this disease. I see the swelling on my breast when I was touch my breast accidentally. Then I did not give it attention and it did not concern me first since it was very small and painless."* ***A 42 years old patient***<br>*"…emmm...I did not get any education about this disease. I did not heard about breast cancer. I saw the changes on my breast while I was sleep once up one a time but I did not think that it will be this kind of disease…I knew a women (one of my husband's sister) who had similar problem with me but she did not go to hospital and it was healed by itself. I was also expect my problem to be healed by itself. I did not think as it would be this bad disease* " **(A 64 years old patient)**<br><br>*"I did not know about cancer in general. I do not know how it will occur and how it is transmitted. I think our major reason for late coming to health facility is our lack of knowledge about the disease. If you go to rural areas you can get a lot of patients with breast problem... most people did not know about the disease "* **A 50 years old patient**<br><br>*"When I heard about cancer, I was frightened because I perceived it as a deadly disease without any treatments… I felt a painless swelling on my breast before 3 years ago but I stayed healthy and it was not have any pain. So I was not think that it could be serious and severe. "* **(A 38 year's old patient said)**

	"I fear surgery, if you come in our community *when a person's breast is removed they do not think that the person will alive. The community has not any awareness about the disease. They have better understanding about HIV, TB and malaria than cancer since they perceive that if someone has developed cancer they think he will be die. Not one believe that cancer has any medical treatments."* **(A 45 years old patient*)***<br>*"This kind of wound is absent in our community. It is not usual…I did not know it before and I did not heard the name itself. The society said me it is 'MITCH (a local name given for any breast swelling in which the community perceived as it is due to exposing of breast to sunlight)… no one educate me about breast cancer before. if a woman developed swelling in her breast, there are herbalists who put a plant leave on the swelling and then it will be healed "* **(A 58 year's old patient said)**<br>*"…I did not know anything about cancer before even I was not think that it has treatments that is why I shouted when they told me that I had cancer."* ***A 36 years old patient***<br>**"***I did not examine my breast. I saw the swelling by accident… I was not going to health facility for check-up of my breast."* **(A68-year-old patient said)**<br>*"My problem was simple at the beginning, no one expect it to be as such ugly. First it was a very small been like swelling and it was painless so I was not consider it as it will be sever disease but the wound becomes burst and tried to produce discharges. Then my neighbor bring me to hospital..."* ***(A 29-year-old patient said)***

**Initial symptom misinterpretation and poor practice of early detection methods**	*"Initially I did not think that it would be cancer. ...No. No. I did not have experience of checking my breast. I saw the swelling when I was washing my body at river. At that time I feel sad and becomes worry but it did not have any pain. I did not do breast self – examination."* **(A 27 years old patient)**<br>*"…I heard the name cancer when people talked but I did not know about it in detail. Not only me did almost all of family not know about cancer. I did not examine my breast. I saw the swelling by accident. When I breast feed my child. There was color change and small swelling initially and later produce bloody discharge then I stopped breast feeding and went to traditional healer…ehhhh… I did not go to health facility for check-up of my breast before."* **(A 42-year-old patient said)**<br>*"Please educate the community about this disease. It is very bad and damages breast. It is killer. I did not wish to other people to have this disease. It disturbed my whole life. when I told my history, ehhhhh… first it was a very small just been like swelling and it was painless so I was not consider it as it will be sever disease but the wound becomes burst and tried to produce discharges. Then my neighbor bring me to hospital. 'Here our main reason for not coming early to hospital is we did not know about cancer…emmmm… if I knew about cancer, I will come early when I saw the swelling early in my breast. But I ignored the initial swelling…"* ***(A 50-year-old patient said)***<br>*"….I know the name cancer but I did not know how it could occur. I did not learnt about cancer. My problem last a long time. I felt a painless swelling on my breast before 3 years but I stayed healthy for the last three years and it was not have any pain. So I was not think that it could be serious and severe…My family also did not think that it will be cancer, they told me as it is self-limited and held by itself… "* **(A 38 year's old patient said)**
**Access to health care facility and transportation related problems**	*"I am not educated. I lived in the rural area. I have five children and I am a farmer. My breast problem starts before two years. But I could not come to health facility early because I did not have money for transportation... There was no also any person who can take family responsibility*

	to grow and to give care for children's. The road is not safe for transportation from my home to health center so most of the time we go through foot to get the nearby health center. it took more than 4 hours but the hospital is too far. **” A 48 year’s old patient said)**<br>*“…there is transportation problem. You cannot get transport easily. You will wait a long time to get transport. I grow three children’s, no one support me economically and I did not have adequate income even to grow my children’s. As a result I could not come early to hospital. I lived with the swelling more than two years since I could not also afford the cost for diagnosis and treatment.”* ***(*A 45 years old patient).**<br>*“… I will tell you the truth. I came to hospital after borrowing money from my young brother. I am a farmer and I have four children's. I am a farmer. Our harvesting system is not satisfactory. We live just hand to mouth. I do not have any money for further treatment after now. I have no any family who can support me. The community support morally but the challenge is how to get money for medical care, transport, bed…..”* **(A 42 years old patient)**
**Cultural and religious related reasons**	*“In my family no one trust on the medical care of cancer rather they trust on GOD and use of holy water and on use of traditional treatments. No one of my family member allowed me to go to operation and to remove my breast rather they told me to use traditional treatment. There are patients who do not want to come to health facility due to fear of losing their breast. There are some people who make you to lose hope. they said you as you could not be improved ”* **(A 36 years old patient*)***<br>*“…When I followed, the swelling becomes increase in size then I feared and I went to traditional healer and he applied herbal medication on it (KEBAW). But it does not improved me rather the swelling becomes burst”.”* ***A 37 years old patient***

	*"I think this is the disease from GOD. .....I saw a small swelling on my right breast but gradually it increases and becomes large. When I follow it becomes grow then I fear and I was going to traditional healer and I applied herbal medication on it. But it does not improved me." (***A 29 year's old patient)**<br>"*…when I saw the swelling in my breast, I became worried and I went to church for pray and took holy water* for a long time. *I did not want to undergo breast surgery. I came know to hospital since the discharge become offensive. I could not sit near to other people because of the smell of the wound". (***A 27 year's old patient)**
**Fear of surgical procedures and lack of trust on medical care**	*"...None of my family member allowed me to go to operation and to remove my breast rather they told me to use traditional treatment. They did not believe that cancer have any medical treatment. They do not trust medical care even they believed that you will be died if you go to hospital and operated. but thy mentioned the experience of some other patients who died after operation and some others who cured applying herbal medication"* **(A 36 years old patient)**<br>*"At first I was not believe that cancer has medical treatment. The community also thinks as cancer do not have any medical treatment. I was giving hope to the traditional treatment and I used it for more than two years but it could not improve me. I know a person who died by cancer but he was treated at hospital. I came now since I do not have any other option. I tried all other options. I used holy water. I am prying day and night. I give it to GOD now "* **(A 37 years old patient)**<br>*".....My family did not allow me to go to hospital and they told me as 'if you go to hospital, they will operate your breast and you could not gave birth after all.' then I was fear and stayed in my*

	home but later I went to use holy water but the swelling became burst and produce offensive discharge. Then I came to hospital since I could not tolerate the pain. " **A 29 years old patient** *"…I fear surgery, ohhh…it is very difficult. I did not believe that a person will be survived after surgery. First it was a very small stone like swelling in my left breast, I was not give it attention at the beginning but late it becomes large and discolored."* **A 64 years old women** *"At first I was not believe that it has medical treatment. The community also thinks as cancer has not any medical treatment. I was giving hope to the traditional treatment and I used it for more than two years but it could not improve me."* **(A 42 years old patient)** *"… I know two patents who were diagnosed with breast cancer and whose breast was removed but after some years they died so the community knows this issue and they did not have a hope on medical treatment."* **(A 55 years old patient)** *".....As you see I am very aged and my neighbors were advised me to not to undergo surgery for my breast rather they advised me to die without losing it…"* ***(A 68 years old patient)***
**Economic hardships and lack of support**	*"My breast problem started before two years. I grow three children's, no one support me economically and I did not have adequate income even to grow my children's. As a result I could not come early to hospital. I lived with the swelling more than two years since I could not afford the cost for diagnosis and treatment. It very difficult to afford the costs for farmers like me. emmmm. It is challenging. The government should make the treatment free"* **(A 45 years old patient).**

	"I was strong before I was diseased. I worked hard. But now I am very week and I felt always the pain. If I came early to hospital, I would be cured but I did not come early. I came after a long time. I stayed using local remedies. My main problem was lack of money for different costs when I went to hospital. I came to hospital after borrowing money from my young brother. I am a farmer and I have four children's. Our harvesting system is not satisfactory. We live just hand to mouth. I do not have any money for further treatment after now. I have no any family who can support me." **(A 42 years old patient)**<br>*"…I had no money for medical cost and all the family responsibility is on me. Even now I have no money for medication and transportation. I did not heard about cancer until the doctor told me. I came from rural area which has not transportation access and we have not money for transport and medical care costs…"* ***A 32 years old patient.***

Participant information

Participant code	Age of participants
**R-1**	**64**
**R-2**	**58**
**R-3**	**27**
**R-4**	**42**
**R-5**	**50**
**R-6**	**38**
**R-7**	**29**
**R-8**	**37**
**R-9**	**36**
**R-10**	**32**
**R-11**	**68**
**R-12**	**45**
**R-13**	**55**
**R-14**	**48**
